# Unveiling shared biomarkers and therapeutic targets between systemic lupus erythematosus and heart failure through bioinformatics analysis

**DOI:** 10.3389/fmed.2024.1402010

**Published:** 2024-06-07

**Authors:** Ting Zhou, Jing Pan, Chenghui Yan, Jing Yuan, Haixu Song, Yaling Han

**Affiliations:** ^1^Department of Cardiology, Union Hospital, Tongji Medical College, Huazhong University of Science and Technology, Wuhan, China; ^2^State Key Laboratory of Frigid Zone Cardiovascular Disease, Cardiovascular Research Institute and Department of Cardiology, General Hospital of Northern Theater Command, Shenyang, China

**Keywords:** hub gene, therapeutic targets, CIBERSORT, systemic lupus erythematosus, heart failure

## Abstract

**Background:**

Systemic lupus erythematosus (SLE) is frequently accompanied by various complications, with cardiovascular diseases being particularly concerning due to their high mortality rate. Although there is clinical evidence suggesting a potential correlation between SLE and heart failure (HF), the underlying shared mechanism is not fully understood. Therefore, it is imperative to explore the potential mechanisms and shared therapeutic targets between SLE and HF.

**Methods:**

The SLE and HF datasets were downloaded from the NCBI Gene Expression Omnibus database. Differentially expressed genes (DEGs) in both SLE and HF were performed using “limma” R package. Gene Ontology (GO) and Kyoto Encyclopedia of Genes and Genes (KEGG) analyses were conducted to analyze the enriched functions and pathways of DEGs in both SLE and HF datasets. Protein–Protein Interaction network (PPI) and the molecular complex detection (MCODE) plugins in the Cytoscape software were performed to identify the shared hub genes between SLE and HF datasets. R package “limma” was utilized to validate the expression of hub genes based on SLE (GSE122459) and HF (GSE196656) datasets. CIBERSORT algorithm was utilized to analyze the immune cell infiltration of SLE and HF samples based on SLE (GSE112087) and HF (GSE116250) datasets. A weighted gene co-expression network analysis (WGCNA) network was established to further validate the hub genes based on HF dataset (GSE116250). Molecular biology techniques were conducted to validate the hub genes.

**Results:**

999 shared DGEs were identified between SLE and HF datasets, which were mainly enriched in pathways related to Th17 cell differentiation. 5 shared hub genes among the common DGEs between SLE and HF datasets were screened and validated, including HSP90AB1, NEDD8, RPLP0, UBB, and UBC. Additionally, 5 hub genes were identified in the central part of the MEbrown module, showing the strongest correlation with dilated cardiomyopathy. HSP90AB1 and UBC were upregulated in failing hearts compared to non-failing hearts, while UBB, NEDD8, and RPLP0 did not show significant changes.

**Conclusion:**

HSP90AB1 and UBC are closely related to the co-pathogenesis of SLE and HF mediated by immune cell infiltration. They serve as promising molecular markers and potential therapeutic targets for the treatment of SLE combined with HF.

## 1 Introduction

Systemic lupus erythematosus (SLE), an intricately systemic autoimmune disease with unknown etiology, presents a greater heterogeneity and complexity due to the multifactorial interplay among various susceptible factors including environment, hormones, and genetics (1). The pathogenesis of SLE involves the production of autoantibodies that target nuclear components, resulting in a wide range of clinical manifestations including skin rashes, arthritis, pleurisy, serositis, and lupus nephritis that can vary over time, making SLE a challenging disease to cure completely, particularly in young women (2). Therefore, investigating the molecular features and mechanisms underlying the onset and progression of SLE holds immense importance in offering novel approaches for successfully preventing, diagnosing, and treating this condition. Heart failure (HF) is a complex chronic clinical syndrome characterized by the deterioration of symptoms and signs resulting from cardiac dysfunction, as well as a significant cause of mortality, morbidity, reduced quality of life, and shortened lifespan (3, 4).

In recent years, the comorbidities of SLE with various other complications have been widely reported including metabolic syndrome, osteoporosis, lupus encephalopathy, and cardiovascular disease (CVD) that encompassed atherosclerosis, HF, and myocardial infarction (MI) (5, 6). Notably, atherosclerosis and increased risk of MI have been acknowledged for numerous years, but limited evidence exists regarding the relationship between SLE and HF (7). A mendelian randomization study showed an association between the genetic susceptibility of SLE and a higher risk of HF (6). Concomitantly, available data suggested that atypical immune responses and chronic inflammation could contribute to hastened atherosclerosis and additional cardiovascular risk factors in individuals with lupus, which result in 5 folds increased risk of HF compared to the general population (7, 8). The risks associated with HF in SLE patients include age, obesity, SLE disease activity index, hyperuricemia, and excessive use of glucocorticoids (7, 9, 10). The pathological mechanisms of HF in SLE patients include myocardial dysfunction (11), left ventricular hypertrophy (12), valvular disease (13), endocarditis (14), myocarditis (15), and pericarditis (16). Despite mounting evidence pointing toward a strong association between SLE and HF, these investigations frequently employ clinical approaches (7, 17), failing to uncover molecular mechanisms occurring at the genetic level. Furthermore, researches on the targeted therapy for patients with concurrent illnesses remain scarce. Consequently, it is imperative to gain deeper insights into the potential mechanisms and shared therapeutic targets involved in both SLE and HF.

In our study, the application of gene microarray technology provided novel insights into the pathogenesis of SLE and HF. Bioinformatics analysis (Gene Ontology (GO) analysis, Kyoto Encyclopedia of Genes and Genes (KEGG) analysis, etc) aided in our comprehension of the genetic factors contributing to SLE and HF development and identified shared core genes and pathways implicated in both SLE and HF based on the datasets downloaded from Gene Expression Omnibus (GEO, https://www.ncbi.nlm.nih.gov/geo/) database. Additionally, we investigated the proportion of immune cell infiltration and the correlation between shared hub genes and immune cell infiltration in the context of SLE and HF, and validated the expression and significance of the most crucial hub genes through weighted gene co-expression network analysis (WGCNA) and real-time polymerase chain reaction (RT-PCR). This comprehensive investigation served as a pioneering study in identifying common biomarkers and therapeutic targets for the comorbidities of SLE and HF, offering valuable insights into the genetic etiology and potential combination therapy strategies for these conditions.

## 2 Materials and methods

### 2.1 Data source

We obtained a high throughput human sequencing data from the GEO database. The data were obtained from the accession number GSE116250 (HF = 37, control = 14) (18), the accession number GSE112087 (SLE = 31, control = 29) (19), the accession number GSE122459 (SLE = 20, control = 6) (20), and the accession number GSE196656 (HF = 3, control = 3) (21). GSE116250 and GSE112087 were used as training datasets. GSE122459 and GSE196656 were used as external validation datasets.

### 2.2 Identification of differentially expressed genes (DEGs)

The DEGs from GSE116250 and GSE112087 were identified using the “limma” R package on normalized count data. The parameters |Log_2_fold change| > 1 and *adj*. *p* < 0.05 were used as the screening criteria for DEGs in GSE116250. The parameters |Log_2_fold change| > 2.5 and *adj*. *p* < 0.05 were used as the screening criteria for DEGs in GSE112087. Moreover, the heatmap and volcano plot of DEGs from the databases were constructed using “heatmap” and “ggplot2” R packages (22). The DEGs were imported to Metascape to perform functional analysis.

### 2.3 GO and KEGG analysis

To uncover the possible biological roles and underlying mechanisms of genes, we employed the R package “clusterProfiler” to analyze the enrichment of KEGG terms pertaining to the target genes. Significance was determined based on KEGG pathways and GO terms encompassing biological processes (BPs), cellular components (CCs), and molecular functions (MFs), with an *adj*. *P* < 0.05 (5).

### 2.4 Immune infiltration analysis

Using the CIBERSORT algorithm, we acquired the proportions of 22 different immune cell types from samples in both GSE116250 and GSE112087. To assess the variations in immune cell levels between disease and control samples, we employed the R package “vioplot” (23).

### 2.5 Correlation analysis between infiltrating immune cells and shared hub genes

The R package “CIBERSORT” was employed to perform the analysis of immune infiltration. Spearman correlation analysis between shared hub genes and infiltrating immune cells was calculated using the R package “corrplot.” Lollipop diagrams were utilized to visualize the correlations between immune cells and hub genes (24).

### 2.6 Construction of WGCNA

We employed the R package “WGCNA” to construct the WGCNA. Initially, hierarchical clustering was executed on the study samples in order to identify any outliers and eliminate the abnormal samples. Next, *β* = 12 was chosen as the soft power parameter for establishing a scale-free network using the pick Soft Threshold function. Subsequently, the adjacency matrix was formulated and transformed into a topological overlap matrix (TOM), followed by the establishment of the gene dendrogram and module color based on the degree of dissimilarity. The “WGCNA” package was then utilized to calculate the correlations between modules and differentially infiltrating immune cells. Modules displaying strong correlation coefficients were regarded as potential candidates associated with differentially infiltrating immune cells and were selected for further analysis. Once the candidate module was chosen, we set the screening criteria for filtering key genes in the candidate module as |MM| (|Module membership|) > 0.8 and |GS| (|gene significance|) > 0.20 (5, 22).

### 2.7 Construction of protein–protein interaction (PPI) network and screening of hub gene

The Interacting Genes Retrieval tools (STRING) database aided in constructing the PPI network (25). The PPI network was subsequently visualized using the Cytoscape software. Identifying significant gene clusters and obtaining hub genes was accomplished by utilizing the molecular complex detection (MCODE) plugins within the Cytoscape software (26).

### 2.8 Mice and MI surgery

The male C57BL/6 mice aged 10 weeks were obtained from Gempharmatech Co., Ltd. (Nanjing, China). Sham and left anterior descending branch (LAD) ligation surgery were performed as described in previously published protocols (27). Temporarily anesthetize mice by inhaling 2% isoflurane. Make a small skin incision on the left chest, expose the heart, and permanently ligate the LAD with 7–0 silk thread. The animals undergoing sham surgery underwent the same procedure, but without ligating the LAD. All animal experiments were performed in accordance with the United Kingdom Animals (Scientific Procedures) Act 1986 and the American Veterinary Medical Association (AVMA) Guidelines for the Euthanasia of Animals (2020). Prior to the study, the research protocol was reviewed and approved by the Medical Ethics Committee of Union Hospital Affiliated to Huazhong University of Science and Technology. We affirmed that this study strictly followed the ARRIVE guidelines (https://arriveguidelines.org).

### 2.9 RNA extraction and RT-PCR

The total RNA from left ventricular tissues was extracted using TRIzol reagent. Subsequently, reverse transcription into cDNA was achieved by utilizing the PrimeScript RT Kit with gDNA Eraser. Afterwards, the mRNA levels of the targeted genes were determined via quantitative PCR using SYBR1 Premix Ex Taq II and normalized to the housekeeping gene *18S* that served as an endogenous internal control. The sequences of mouse species primers used in our study are as follows:

*Heat shock protein 90 alpha family class B member 1 (HSP90AB1)*: Forward: 5′-TGGCTGAGGACAAGGAGAACT AC-3′; Reverse: 5′-GAGAGGCGGCGTCGGTTAG-3′.

*Ribosomal protein lateral stalk subunit P0 (RPLP0)*: Forward: 5′-AGCTGCTGCCACCACTGC-3′; Reverse: 5′-TCATCTGATTCC TCCGACTCTTCC-3′.

*Ubiquitin C (UBC)*: Forward: 5′-CCACCAAGAAGGTCAAAC AGGAAG-3′; Reverse: 5′-TCACACCCAAGAACAAGCACAAG-3′.

*Ubiquitin B (UBB)*: Forward: 5′-ACCTGGTCCTCCGCCTGAG-3′; Reverse: 5′- ATGCCCTCTTTATCCTGGATCTTGG-3′.

*Neural precursor cell-expressed developmentally downregulated 8 (NEDD8)*: Forward: 5′-TGGCATCACATATCCTCTCACTCTC-3′; Reverse: 5′- CCCACCAGTAGACACACAAGATTG-3′.

*18S*: Forward: 5′-ACATCATCCCTGCATCCACT-3′; Reverse: 5′- GGGAGTTGCTGTTGAAGTCA-3′.

### 2.10 Statistical analysis

Bioinformatics statistical analysis was performed using R software v4.3.1 (http://www.r-project.org/). The expression of the hub genes was analyzed using the “Wilcox. Test,” *adj*. *p* < 0.05 was considered statistically significant. Molecular biology experimental data were presented as the mean ± standard error of the mean (SEM) of at least 3 independent experiments. Differences between the two groups were evaluated using the unpaired Student’s two-tailed t-test. Normal distribution of the data was analyzed using a Shapiro Wilk test. All data were analyzed using GraphPad Prism 8.3 (GraphPad Software, San Diego, CA). *p < 0.05; **p < 0.01; ns. indicates no significance between the 2 indicated groups.

## 3 Results

### 3.1 Data processing and quality control

Human training datasets (HF dataset GSE116250 and SLE dataset GSE116250) and human validation datasets (HF dataset GSE122459 and SLE dataset GSE196656) were retrieved and downloaded from the GEO database. The quality control of training datasets was performed through Principal Component Analysis (PCA) clustering analysis. It was observed that all samples from the HF and SLE datasets were well separated, suggesting high data quality (Supplemental Figure S1).

### 3.2 Identification of DEGs in SLE and HF datasets

**Figure 1:**
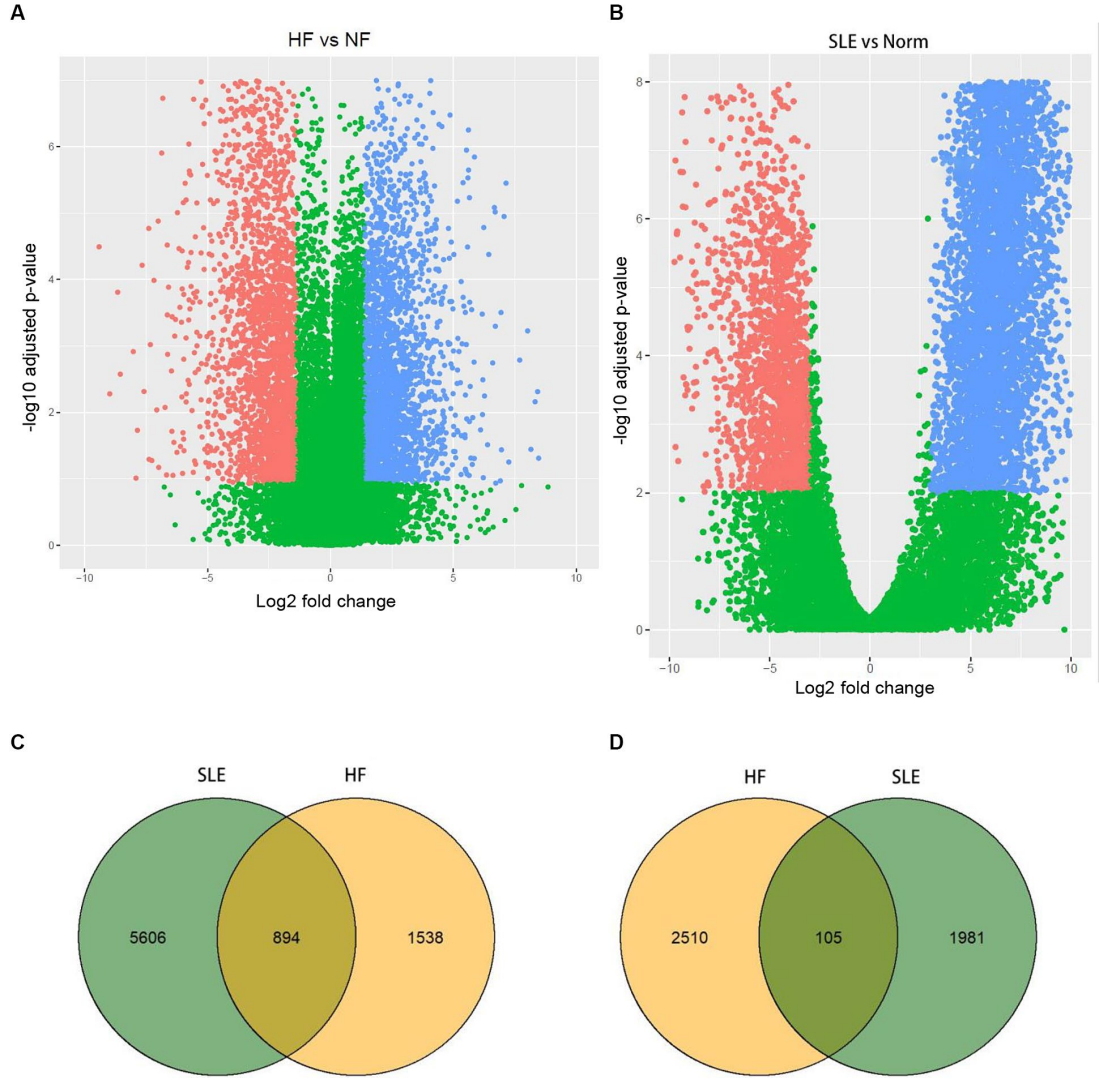
Identification of DEGs in both HF and SLE datasets. **(A)** Volcano map showing the DEGs in HF dataset. **(B)** Volcano map showing the DEGs in SLE dataset. **(C)** Venn plot showing the upregulated DEGs in HF and SLE datasets. **(D)** Venn plot showing the downregulated DEGs in HF and SLE datasets.

In order to identify significant genes in the HF dataset, we applied a screening criterion of |log_2_FC| > 1 and *adj*. *p* < 0.05. Similarly, for the SLE dataset, we used a screening criterion of |log_2_FC| > 2.5 and *adj*. *p* < 0.05. Volcano maps were subsequently created for the HF and SLE datasets. The HF dataset showed 1,538 upregulated genes and 2,510 downregulated genes, while the SLE dataset showed 5,606 upregulated genes and 1,981 downregulated genes (Figures 1A,B). Venn diagrams showed a total of 894 overlapping upregulated genes and 105 overlapping downregulated genes between the HF and SLE datasets (Figures 1C,D).

### 3.3 The function enrichment analysis of HF and SLE datasets

**Figure 2:**
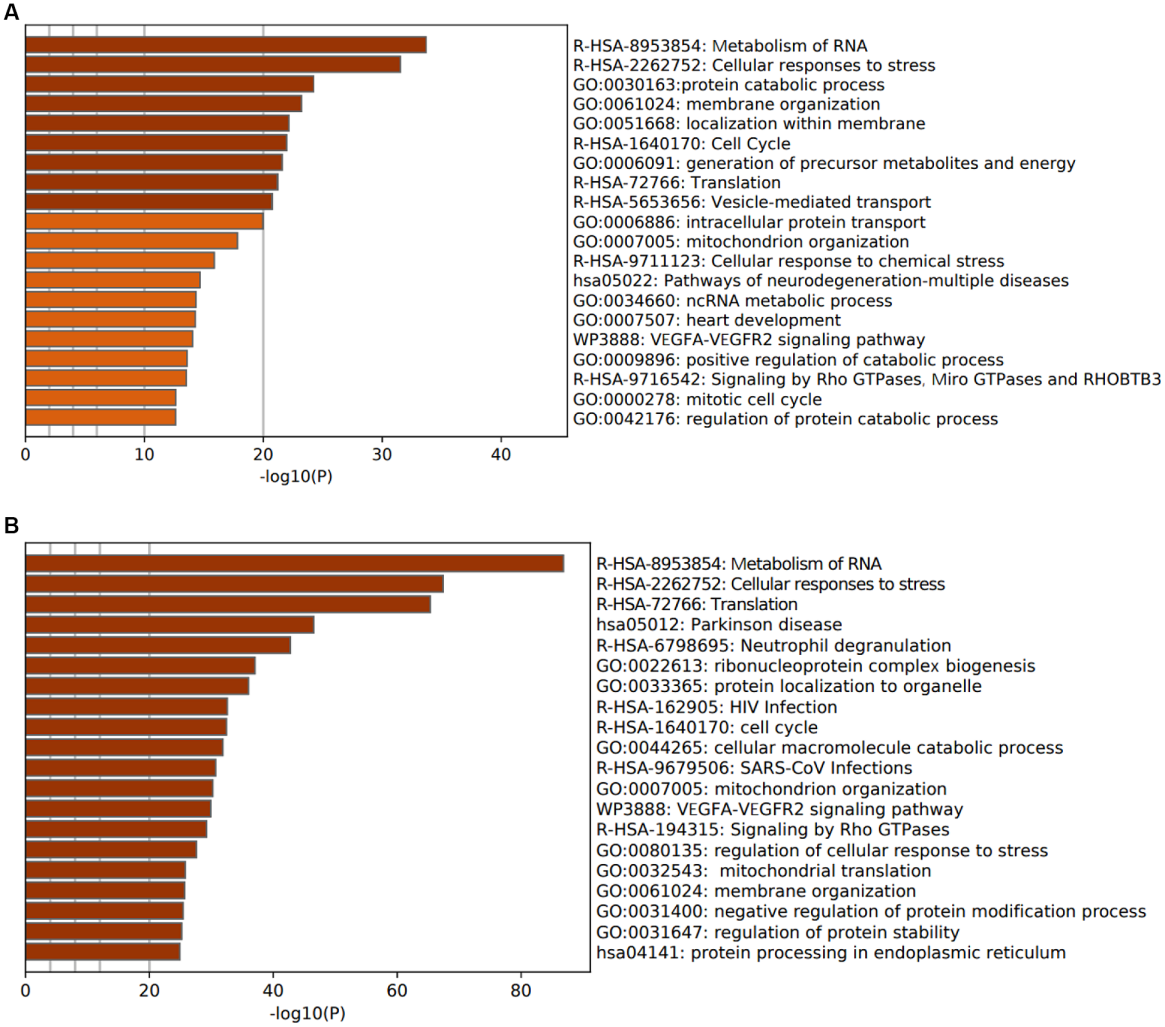
GO analysis for DEGs in both HF and SLE datasets. **(A)** GO analysis for the DEGs in HF dataset. **(B)** GO analysis for the DEGs in SLE dataset.

**Figure 3:**
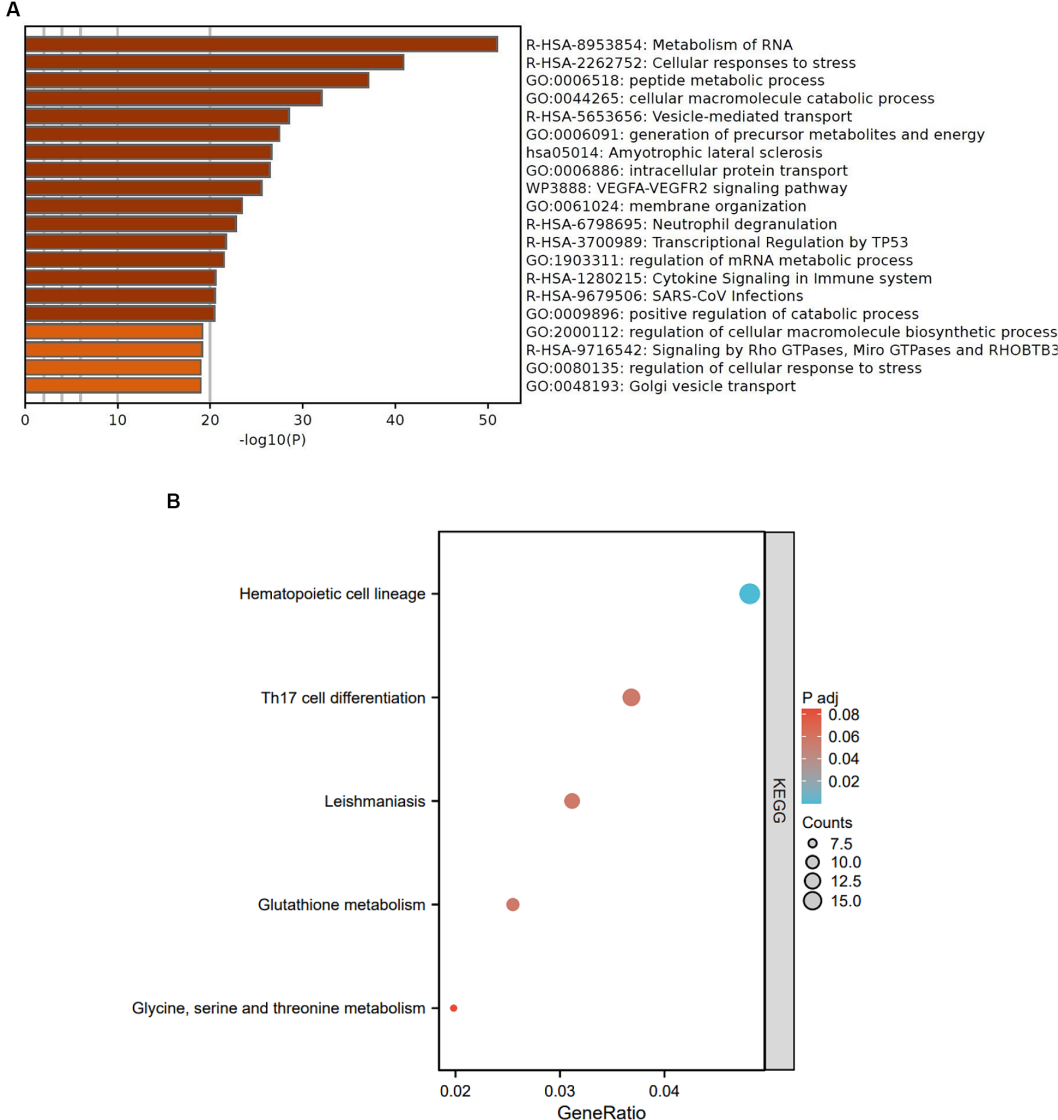
GO and KEGG analysis for the common DEGs between HF and SLE dataset. **(A)** GO analysis for the common DEGs between HF and SLE dataset. **(B)** KEGG analysis for the common DEGs between HF and SLE dataset.

To investigate the functions and pathways associated with DEGs in SLE and HF datasets, functional enrichment analysis was performed using GO analysis. The results revealed that the DEGs in HF dataset were mainly enriched in pathways related to metabolism of RNA, cellular responses to stress, protein catabolic process, translation, and positive regulation of protein catabolic process (Figure 2A). Similarly, the DEGs in SLE dataset were prominently enriched in pathways related to metabolism of RNA, cellular responses to stress, regulation of protein stability, cellular macromolecule catabolic process, protein localization to organelle, and mitochondrial organization (Figure 2B). Furthermore, GO analysis of the common DEGs between HE and SLE datasets were predominantly enriched in pathways related to metabolism of RNA, cellular responses to stress, peptide metabolic process, cellular macromolecule catabolic process, positive regulation of catabolic process, and vesicle-mediated transport (Figure 3A). Concomitantly, KEGG analysis of the common DGEs between HE and SLE datasets were predominantly enriched in pathway related to Th17 cell differentiation, supporting the notion that immune system disorders might serve as a shared pathogenic factor for both HF and SLE (Figure 3B).

### 3.4 Screening of the hub genes between HF and SLE datasets

**Figure 4:**
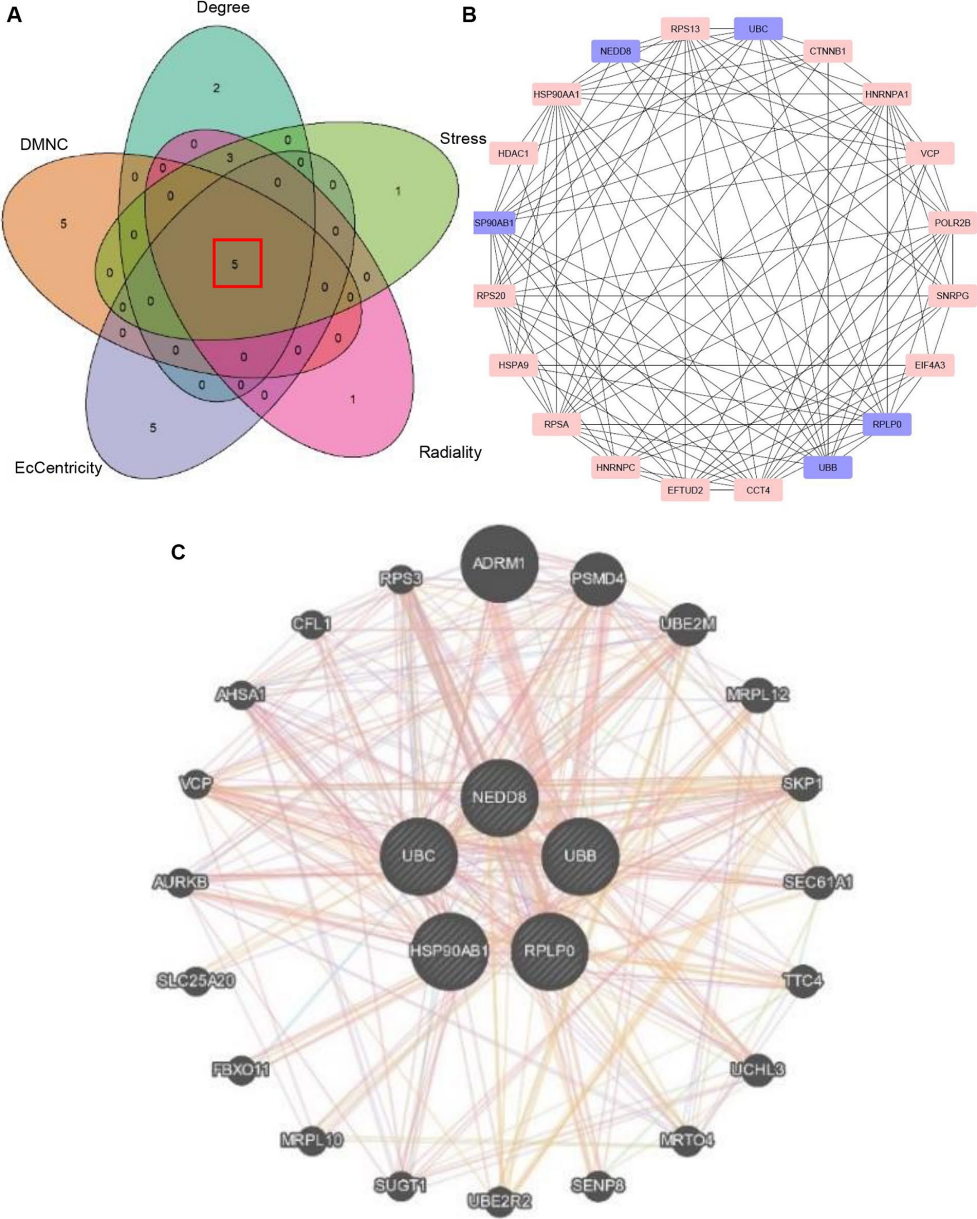
Identification of hub genes. **(A)** Venn plot showing 5 overlapping hub genes based on 5 algorithms. **(B)** PPI network visualizing the hub genes. **(C)** The co-expressed genes of 5 hub genes.

The common 999 DEGs between HF and SLE datasets were imported into the STRING database to construct a PPI network. Afterwords, the top 10 common DGEs with the highest ranking were identified based on 5 algorithms (Degree, DMNC, Eccentricity, Radiance, and Stress) from MCODE plugins in the Cytoscape software (Supplementary Table S1). Moreover, the 5 overlapping hub genes including *NEDD8*, *UBC*, *HSP90AB1*, *UBB*, and *RPLP0*, were identified using Venn plots (Figure 4A) and displayed in the PPI network (Figure 4B). Additionally, the co-expressed genes associated with 5 shared hub genes were further analyzed using GeneMANIA (28), among which the most significant genes with the highest ranking associated with these hub genes were adhesion regulating molecule 1 (ADRM1), proteasome 26S subunit, non-ATPase 4 (PSMD4), ubiquitin-conjugating enzyme E2M (UBE2M), mitochondrial ribosomal protein L7/L12 (MRPL12), and S-phase Kinase-Associated Protein 1 (SKP1) (Figure 4C).

### 3.5 Validation of the shared hub genes in HF and SLE datasets

**Figure 5:**
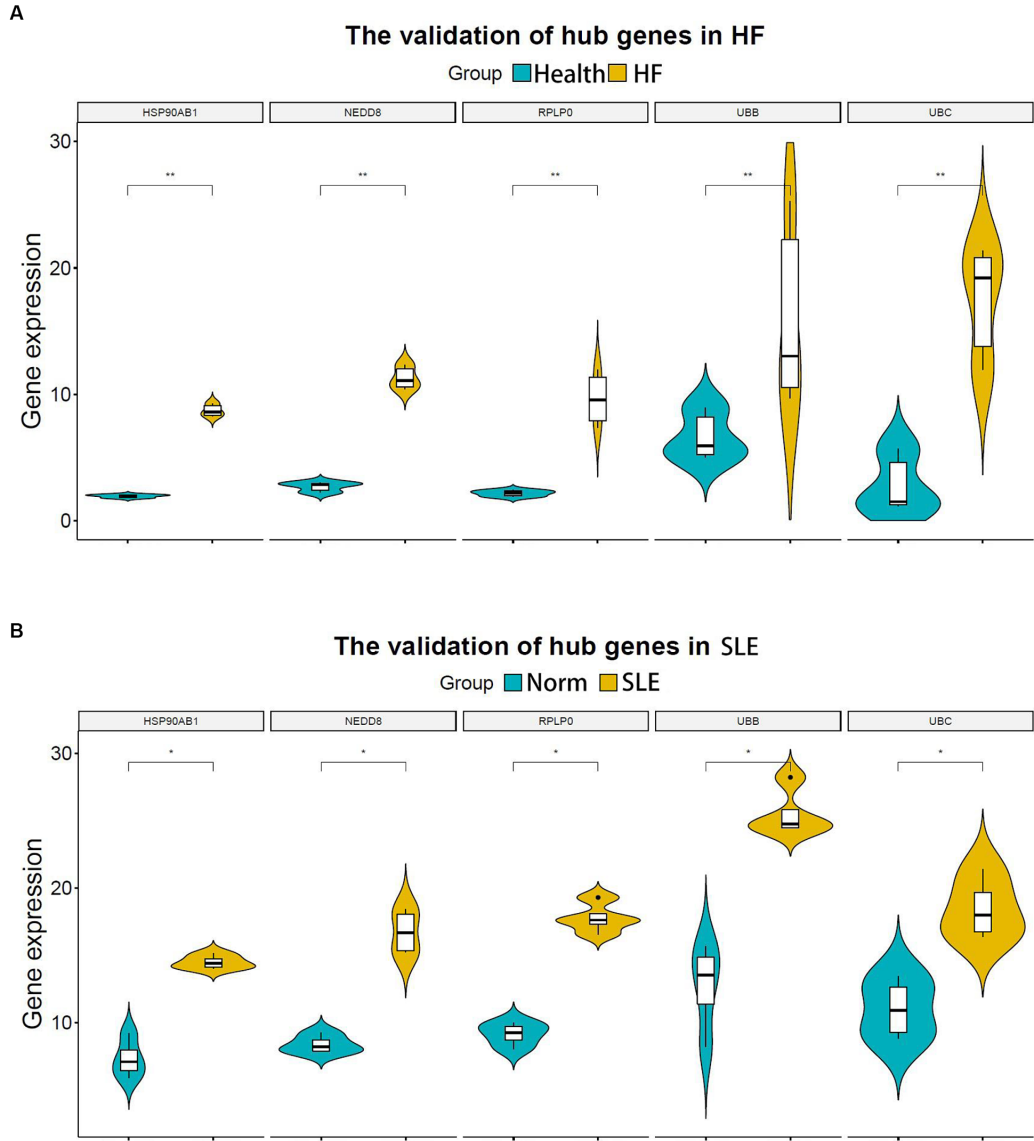
Validation of hub genes in both HF and SLE datasets. **(A)** 5 hub genes validated in the HF dataset (GSE196656). **(B)** 5 hub genes validated in the SLE dataset (GSE122459). *P < 0.05; **P < 0.01.

In the subsequent analysis, we validated the expression levels of the 5 shared hub genes in patients with lupus and patients with HF using the human SLE dataset (GSE122459) and the human HF dataset (GSE196656) respectively. The results from the violin plots indicated an increase in the expression of 5 hub genes in both patients with HF (Figure 5A) and patients with lupus (Figure 5B) in comparison to those in normal individuals, suggesting that these 5 hub genes may be co-pathogenic hub genes for both HF and SLE patients.

### 3.6 Immune cell infiltration in samples of the HF and SLE datasets

**Figure 6:**
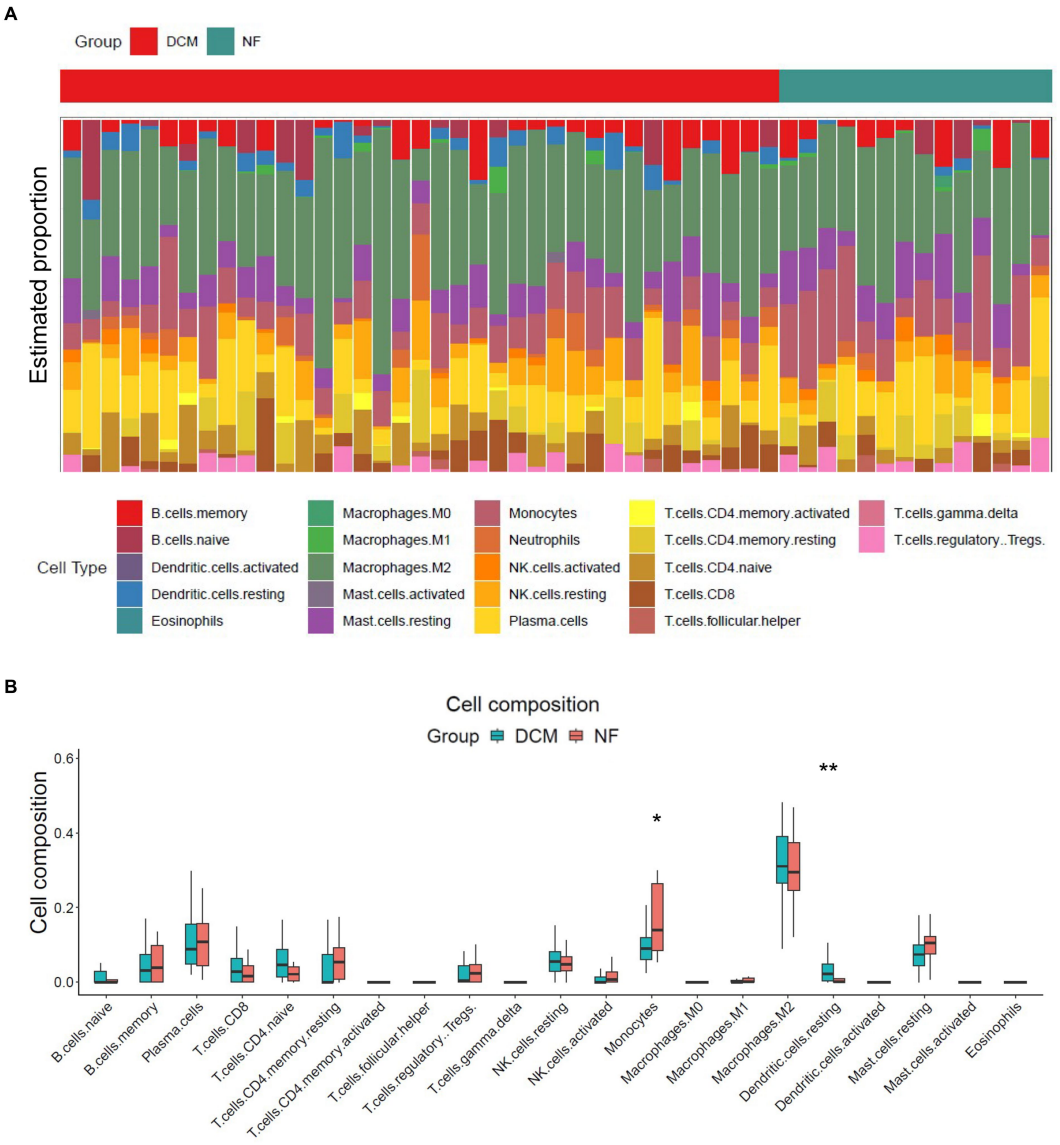
Immune cell infiltration analysis for HF dataset. **(A)** The proportion of 22 immune cells in HF samples. **(B)** The immune cell infiltration in HF samples.*P < 0.05; **P < 0.01.

**Figure 7:**
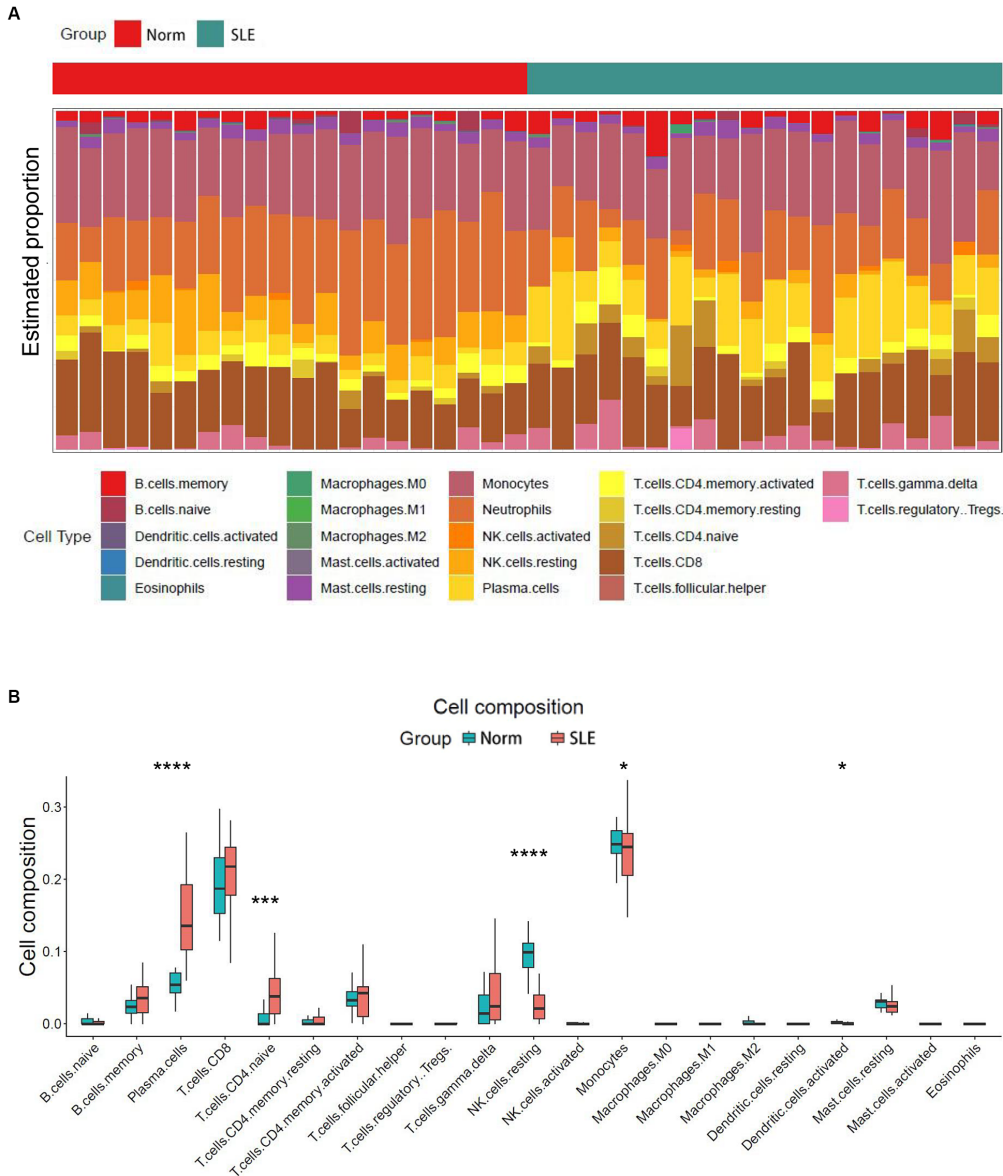
Immune cell infiltration analysis for SLE dataset. **(A)** The proportion of 22 immune cells in SLE samples. **(B)** The immune cell infiltration in SLE samples. *P < 0.05; ***P < 0.001; ****P < 0.0001..

The release of pro-inflammatory cytokines and infiltration of immune cells are characteristic features of HF (29, 30). Similarly, SLE is also characterized by the infiltration of various immune cells (31, 32). Therefore, we used CIBERSORT algorithm to investigate the extent of immune cell infiltration in these two distinct conditions based on HF dataset (GSE116250) and SLE dataset (GSE112087). The HF datasets showed a significant increase in monocytes and a decrease in dendritic cells in HF patients compared to those in control group (Figure 6), which was consistent with the findings in the SLE dataset regarding dendritic cells. Additionally, the SLE dataset revealed an increase in plasma cells and CD4+ naive cells, as well as a decrease in monocytes and NK cells (Figure 7). These findings suggested a potential shared pathogenesis based on immune cell infiltration between HF and SLE, particularly dendritic cells infiltration.

### 3.7 Correlation between the shared hub genes and immune cell infiltration

**Figure 8:**
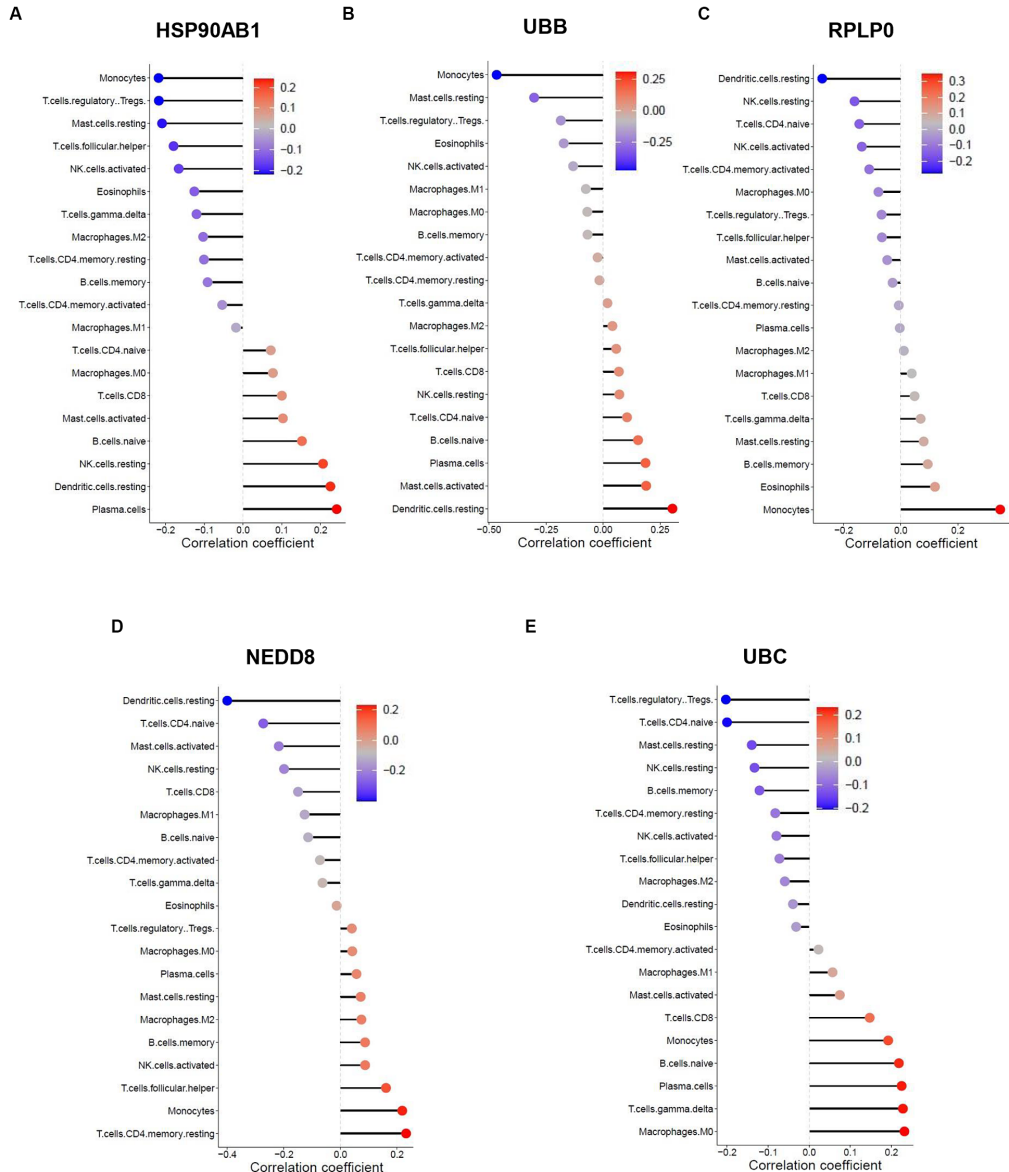
Spearman correlation analysis between the hub genes and immune cell infiltration in HF dataset. The correlation between **(A)** HSP90AB1, **(B)** UBB, **(C)** RPLP0, **(D)** NEDD8, **(E)** UBC and immune cell infiltration in HF dataset.

**Figure 9:**
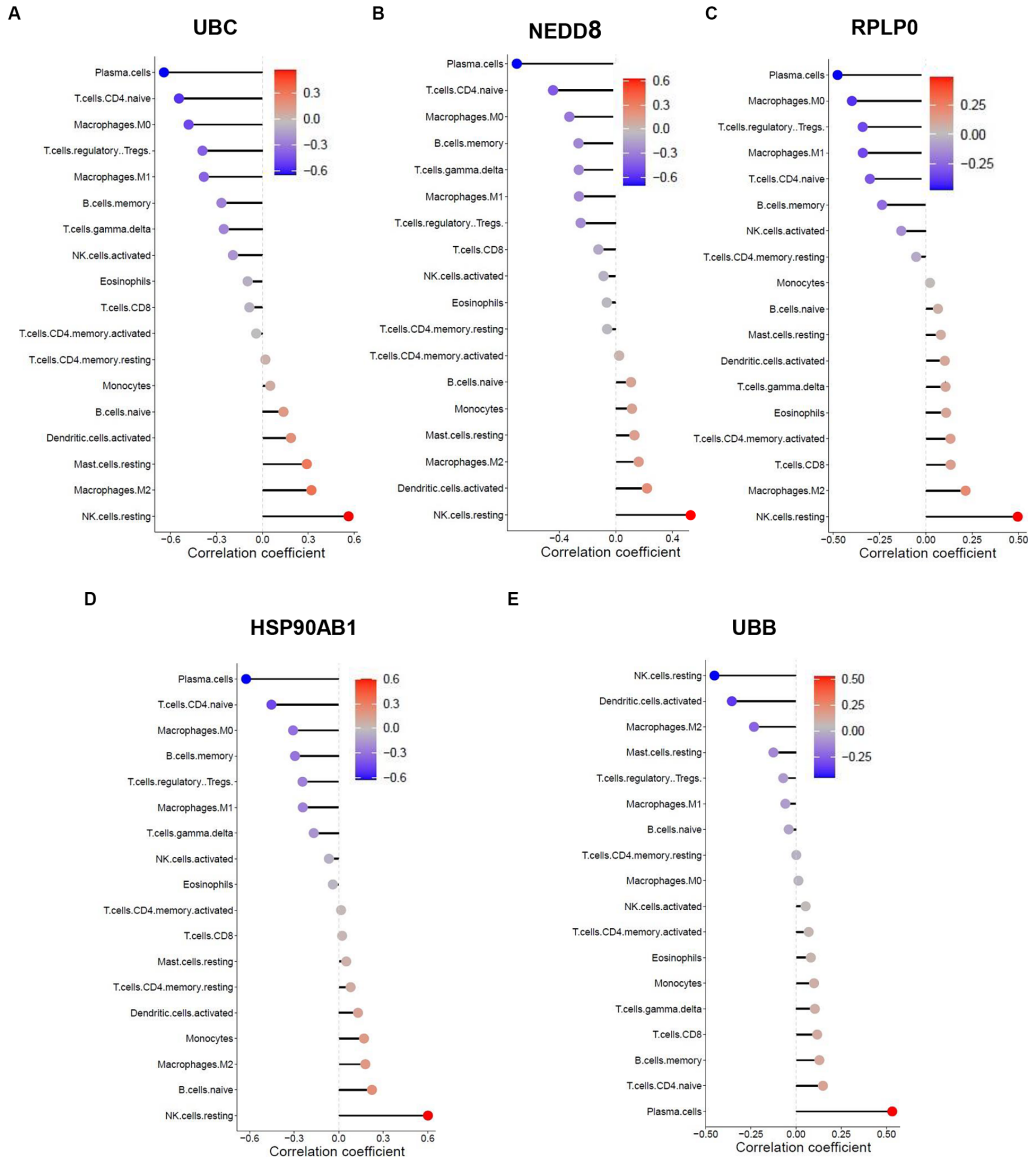
Spearman correlation analysis between the hub genes and immune cell infiltration in SLE dataset. The correlation between **(A)** UBB, **(B)** NEDD8, **(C)** RPLP0, **(D)** HSP90AB1, **(E)** UBB and immune cell infiltration in SLE dataset.

The similarity in immune cell composition constitutes solely one facet of the mutual pathogenesis shared by both HF and SLE. It remains imperative to confirm whether these 5 shared hub genes are involved in immune infiltration, identify the specific immune cells they are associated with, and determine their common characteristics. Consequently, we performed the Spearman correlation analysis to examine the correlation between these 5 shared hub genes and immune cell infiltration based on HF and SLE datasets. The results showed that HSP90AB1 and UBB were negatively correlated with monocytes and positively correlated with dendritic cells (Figures 8A,B), while NEDD8, RPLP0, and UBC showed a negative correlation with dendritic cells and a positive correlation with monocytes in the HF dataset (Figures 8C–E). On the other hand, HSP90AB1, NEDD8, RPLP0, and UBC were found to be positively correlated with both monocytes and dendritic cells (Figures 9A–C). While UBB showed a positive correlation with monocytes and a negative correlation with dendritic cells in the SLE dataset (Figures 9D,E). In general, a stable and positive correlation existed between hub genes (NEDD8, RPLP0, and UBC) and monocytes, as well as between HSP90AB1 and dendritic cells in the HF and SLE datasets.

### 3.8 Validation of the shared hub genes by WGCNA

**Figure 10:**
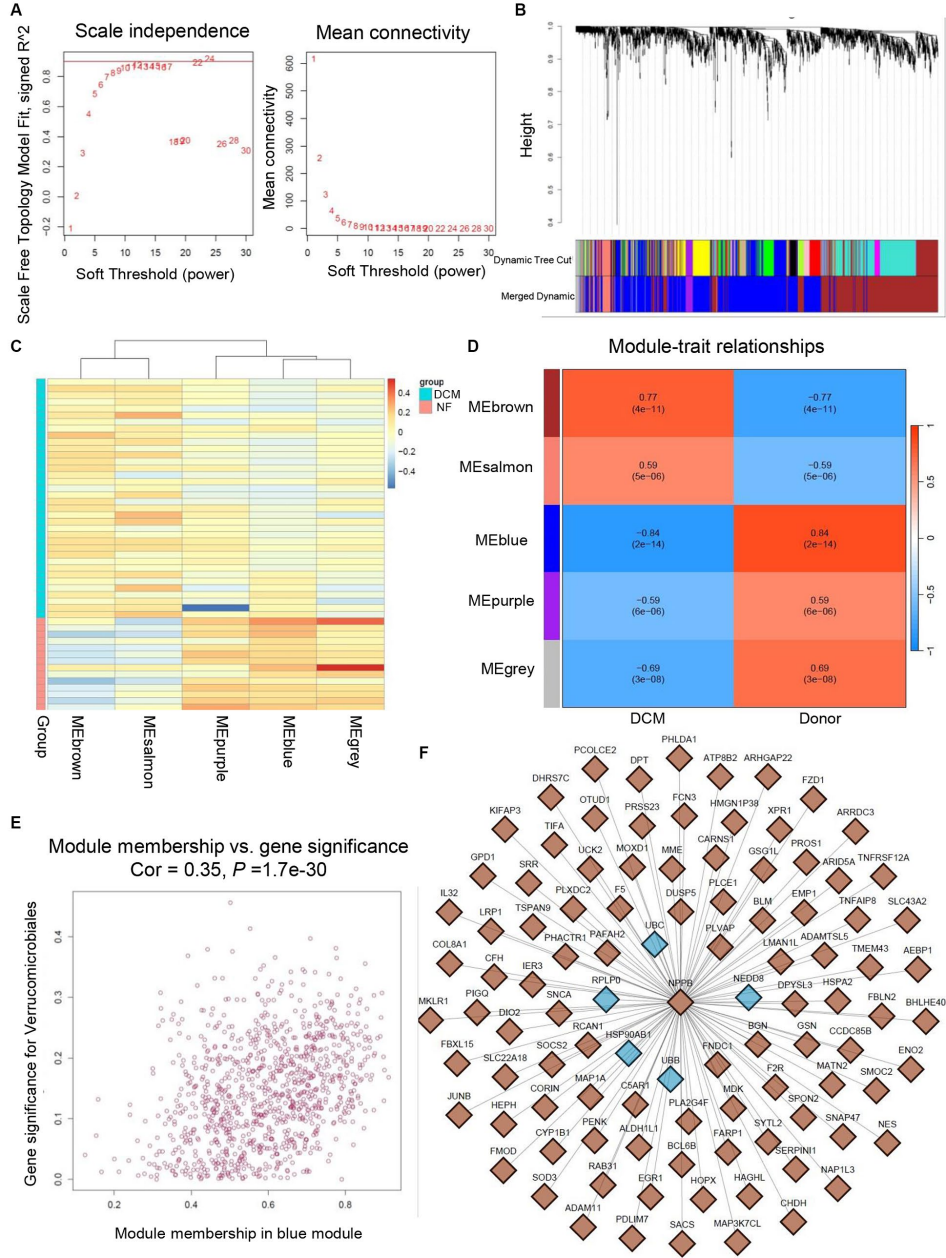
Validation of hub genes through WGCNA. **(A)** Determination of soft threshold power for GSE116250 dataset. **(B)** Origin and merged modules displaying under the clustering tree for GSE116250 dataset. **(C)** Heatmap showing the correlation between module eigengenes and the occurrence of HF. **(D)** Heatmap showing the correlation between module eigengenes and the occurrence of HF. **(E)** Scatter plot of the eigengenes in MEbrown module. **(F)** Protein interaction map showing the location of hub genes within the MEbrown module.

To further verify the significance of hub genes in HF and SLE datasets, we initially created a gene co-expression network using the R package “WGCNA” based on the expression levels of genes in the GSE116250 dataset. Afterwards, cluster analysis was conducted for all the samples within the dataset except for the outlier sample dilated cardiomyopathy (DCM) 28 (Supplemental Figure S2). Additionally, a scale-first network with *β* = 12 as the soft threshold power was successfully established (Figure 10A). Next, the average linkage and Spearman correlation coefficients were calculated to construct a hierarchical clustering tree in which each leaf represented a specific gene and each branch represented a specific module that encompassed all genes exhibiting comparable expression levels (Figure 10B). Finally, we consolidated the functionally equivalent modules into a single large module, resulting in a total of 5 modules, among which the MEbrown module showed the highest correlation with HF (Figures 10C,D). Therefore, eigengenes within the MEbrown module were selected for the scatter map and imported into Cytoscape software to construct a protein interaction map (Figure 10E). Additionally, 5 shared hub genes were found to be located in the central part of this module, further confirming their significant role in the pathogenesis of HF (Figure 10F).

### 3.9 Validation of the shared hub genes by RT-PCR

**Figure 11:**
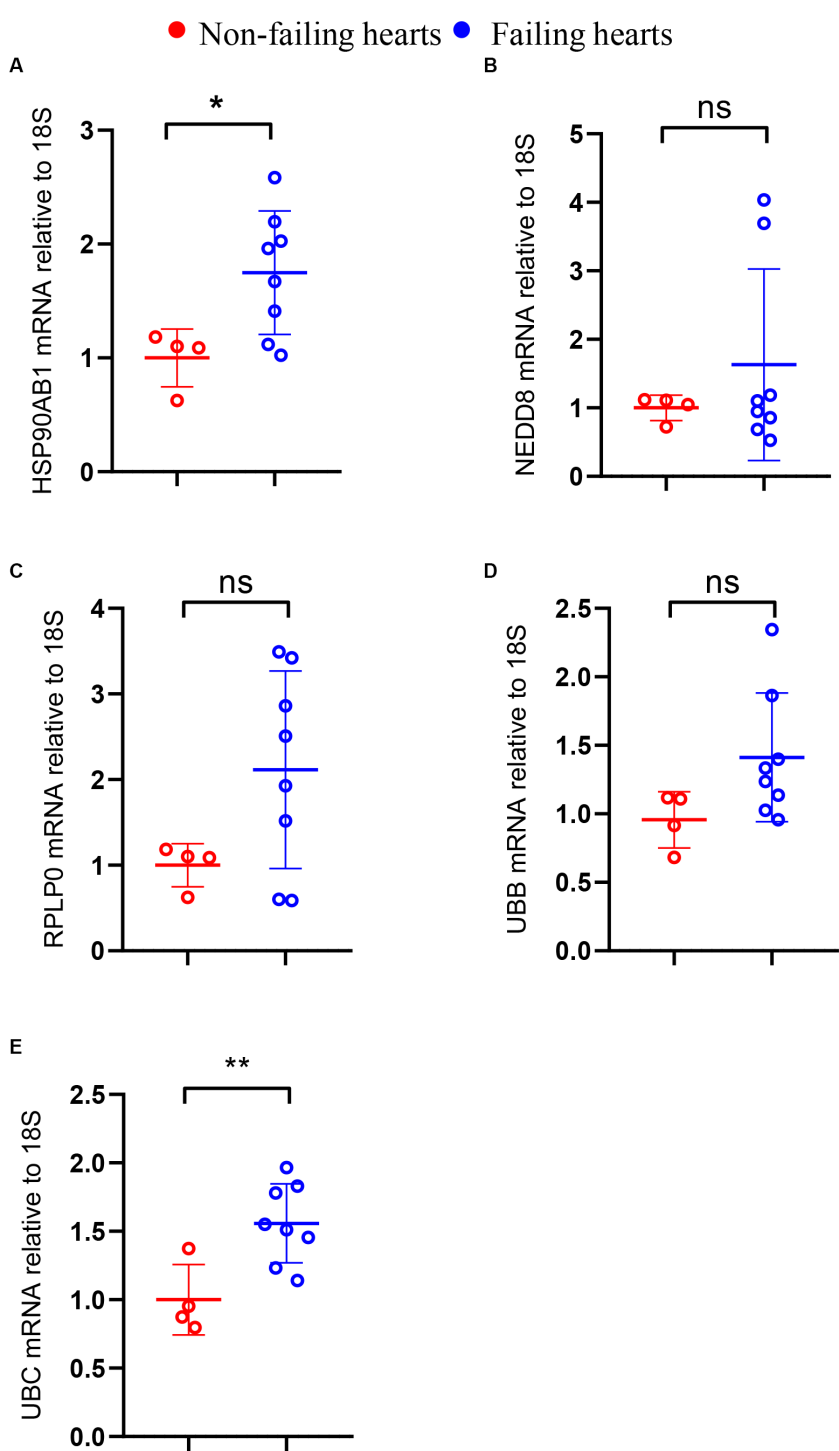
Validation of hub genes through RT-PCR. The mRNA levels of HSP90AB1 **(A)**, NEDD8 **(B)**, RPLP0 **(C)**, UBB **(D)**, and UBC **(E)** between non-failing and failing hearts. *P < 0.05; **P < 0.01; ns. indicates no significance between the 2 indicated groups.

Considering the limited sample size of the validation dataset (GSE196656), a HF model was constructed by subjecting 10-week-old C57BL/6 wild type mice to MI surgery for 3 weeks to further validate the expression of hub genes in failing hearts in comparison to that in non-failing hearts. RT-PCR analysis confirmed a high expression level of HSP90AB1 and UBC in the hearts of MI mice compared to that in sham mice (Figures 11A,E), while the expression of UBB, RPLP0, and NEDD8 showed no obvious change between the two groups (Figures 11B–D).

## 4 Discussion

SLE is an autoimmune condition that exhibits both CVD and non-CVD complications. The association between SLE and CVD, such as accelerated atherosclerosis and increased risk of MI that contribute to heightened risks of HF in this population, is well-established (12, 16, 33). However, limited research has been conducted to verify the specific link between SLE and HF. Moreover, due to advancements in therapies in recent decades, the overall survival rate of SLE patients has improved. However, their mortality rate induced by HF has remained largely constant (8). Therefore, it is imperative to carry out rigorous investigations that focus on the exact mechanism of interaction between SLE and HF to optimize HF outcomes in SLE patients.

This study aimed to explore the genetic and molecular resemblances between SLE and HF. The objective was accomplished by carrying out a thorough bioinformatics analysis to detect the common hub genes and underlying mechanisms shared by SLE and HF. Our analytical approach began by comparing the DEGs between SLE and HF using the human HF dataset (GSE116250) and the human SLE dataset (GSE112087). We then performed a function enrichment analysis on the DEGs in HF and SLE datasets respectively, as well as on the common DEGs shared by HF and SLE datasets. Furthermore, we utilized 5 algorithms from the Cytoscape plugins (MCODE) to identify 5 hub genes (HSP90AB1, NEDD8, RPLP0, UBB, and UBC) among the 999 common DEGs and further validated the expression of 5 hub genes in both SLE and HF samples using the human SLE (GSE122459) dataset and the human HF (GSE196656) dataset. Additionally, we conducted an investigation into the immune cell infiltration in both SLE and HF samples, while also examining the correlation between the shared 5 hub genes and immune cell infiltration using CIBERSORT algorithm. Notably, we discovered that these 5 hub genes were strongly linked to immune-related monocytes and dendritic cells in both SLE and HF. Furthermore, we identified the MEbrown module that displayed the strongest association with the DCM phenotype by WGCNA, and validated that the hub genes were within the core position of MEbrown module. Additionally, we validated the increased expression of HSP90AB1 and UBC in the mice failing hearts compared to non-failing hearts by RT-PCR.

HSP90AB1 is a member of the large family of HSPs that function as molecular chaperone, which binds to client proteins including kinases, ubiquitin ligases, and transcription factors, to support proper protein folding and maintain protein stability, especially after exposure to various kinds of cellular stress (34). Numerous bioinformatics studies suggest that HSP90AB1 serves as a potential biomarker of end-stage DCM induced HF (35). Additionally, HSP90AB1 interacts with transforming growth factor-β receptor on the plasma membrane surface of cardiac fibroblast, facilitating pressure overload-induced cardiac remodeling and HF (36). Concomitantly, HSP90AB1 is reported to be a PANoptosis-related gene that contributes to immune dysfunction in SLE (34). Copy number variations of HSP90AB1 are associated with a high risk of SLE, making it an ideal diagnostic biomarker for SLE (37). Our research reveals that HSP90AB1 levels are elevated in both HF and SLE. Additionally, the association between HSP90AB1 and HF is validated through both WGCNA and RT-PCR, suggesting HSP90AB1 as a potential link between HF and SLE and proposing a theoretical immunotherapeutic target for patients with both conditions.

NEDD8 exhibits extensive expression in various tissues and cell types, with the highest levels in cardiac and skeletal muscle tissues. The homology of NEDD8 is 100% among rats, mice, and humans, highlighting its conserved function within eukaryotic cells (38). Similar to ubiquitination, the post-translational modification mediated by NEDD8 is known as neddylation that plays a crucial role in maintaining cardiac homeostasis and preserving the structural and functional integrity of the heart (39). Additionally, NEDD8-mediated neddylation plays a crucial role in sustaining the homeostasis of T cells and facilitating the advancement of SLE. The inhibition of neddylation can trigger the programmed cell death of T cells in patients with SLE, thereby markedly enhancing the course of SLE progression (40). Our study demonstrates that there is an increase in NEDD8 levels in both HF and SLE. Moreover, while WGCNA confirms the close link between NEDD8 and HF, RT-PCR does not provide supporting evidence. Hence, the role of NEDD8 as a contributing factor to both HF and SLE remains inconclusive.

RPLP0, a ribosomal protein, encodes the large P0 subunit that constitutes a crucial part of the 60S subunit (41). RPLP0 shows stable expression levels across different heart cavities and disease conditions, making it an optical reference gene in gene expression analysis (42, 43). RPLP0 is upregulated in maladaptive remodeling of the right ventricle and regulates the progression of maladaptive hypertrophic response through facilitating ribosomal protein synthesis (44). Meanwhile, the levels of anti RPLP0 antibodies are elevated in patients with SLE compared to the healthy control group, contributing to the development of lupus lesions associated with SLE (45). Our study reveals an elevation in RPLP0 levels in both HF and SLE. Although WGCNA validates the association between RPLP0 and HF, RT-PCR results do not demonstrate a rise in RPLP0 expression in HF samples. Consequently, the involvement of RPLP0 as a potential factor in both HF and SLE remains inconclusive.

UBB, a highly conserved protein, encodes ubiquitin, which plays a critical role in directing cellular proteins toward degradation by the 26S proteasome (46). UBB is elevated in the heart of rats under unloading, pressure overloading, and hypoxic conditions, serving as a potential target to reverse pathologic processes during cardiac remodeling (47). Additionally, UBB functions as an oncogene linked to HF by promoting the proliferation of cardiomyocytes (48). Nevertheless, the research on UBB in SLE is limited at present, which makes our research on the diagnostic and therapeutic value of UBB in SLE and HF meaningful. Our research shows that UBB levels are elevated in both HF and SLE. Although WGCNA verifies the strong association between UBB and HF, RT-PCR fails to validate this connection. Therefore, the involvement of UBB in the development of both HF and SLE is still uncertain.

UBC encodes a precursor polyubiquitin protein and plays a vital role in maintaining the protein homeostasis (49). Analysis of the PPI network indicates that UBC may have a crucial involvement in promoting cardiac inflammation and fibrosis during the cardiac injury induced by angiotensin II (50). However, reports on UBC in SLE remain scarce. Our study shows that levels of UBC are increased in both SLE and HF. Moreover, the correlation between UBC and HF is confirmed using both RT-PCR and WGCNA, indicating UBC as a co-pathogenic gene for both SLE and HF, and proposing potential therapeutic approaches for individuals with concurrent conditions.

Previous studies have demonstrated the significant role of immune cell infiltration in the progression of both SLE and HF (29, 32, 51). Furthermore, HSP90AB1 shows increased expression in HF mice and serves as the hub gene and immunotherapy targets in HF, which is essential in the oxidative stress and immune infiltration of HF (52). The meta-analysis findings show all gene pathways focused on the UBC gene are linked to immune response and inflammation (53). In this study, we investigated the composition of immune cell infiltration in SLE and HF, which enhanced our understanding of its role in both conditions. Our analysis reveals a reduction in the presence of dendritic cells, which could potentially contribute to the pathogenesis and progression of SLE in combination with HF and should be a focus of further research. In addition, our analysis showed a consistent correlation between UBC and monocytes, as well as between HSP90AB1 and dendritic cells in the both the HF and SLE datasets, indicating that HSP90AB1 and UBC were possibly involved in the shared pathogenesis of HF and SLE through regulating immune cell infiltration.

There are still some limitations in our study that need to be addressed. Firstly, the validation dataset (GSE196656) we utilized had a limited sample size. In order to enhance the validity of our findings, it would be beneficial to obtain larger datasets pertaining to HF and additional datasets pertaining to the comorbidity between SLE and HF. Secondly, further investigation is required to determine the precise mechanisms behind the association between immune cell infiltration and shared hub genes. Consequently, it is imperative to further verify our results through both *in vivo* and *in vitro* experiments.

## 5 Conclusion

Our study not only presents a unique approach for identifying the shared hub genes (HSP90AB1 and UBC) in peripheral blood from patients with SLE and left ventricular tissue from patients with HF, respectively, but also offers theoretical strategies and new insights into the shared pathogenic mechanisms and potential combination therapies for individuals with both SLE and HF.

## Data availability statement

The original contributions presented in the study are included in the article/Supplementary material, further inquiries can be directed to the corresponding authors.

## Ethics statement

The animal study was approved by the Medical Ethics Committee of Union Hospital Affiliated to Huazhong University of Science and Technology. The study was conducted in accordance with the local legislation and institutional requirements.

## Author contributions

YH: Formal analysis, Data curation, Writing – review & editing, Writing – original draft. TZ: Writing – review & editing, Writing – original draft. JP: Writing – original draft, Validation, Software, Resources, Funding acquisition. CY: Writing – original draft, Visualization, Validation, Investigation, Formal analysis, Data curation, Conceptualization. JY: Writing – review & editing, Writing – original draft. HS: Writing – review & editing, Writing – original draft, Resources, Funding acquisition.
