# Supplementary material for: Unveiling shared biomarkers and therapeutic targets between systemic lupus erythematosus and heart failure through bioinformatics analysis
Source: Front Med (Lausanne). 2024 Jun 7;11:1402010. doi: 10.3389/fmed.2024.1402010 (PMC11190381; doi:10.3389/fmed.2024.1402010)
Supplement: Supplementary file 1 [file Data_Sheet_1.PDF]

Supplementary Information

Supplemental Table S1. The top 10 common DGEs with the highest ranking between SLE (GSE112087) and HF (GSE116250) datasets were identified.

Degree	DMNC	EcCentricity	Radiality	Stress
CTNNB1	CCDC47	ACTN4	HSP90AA1	HSP90AA1
EFTUD2	COX7B	BID	UBB	UBB
HDAC1	HTATIP2	CAT	HDAC1	PKM
HNRNPA1	MRPL28	GRB2	RPSA	HDAC1
HSP90AA1	MRPL34	GSK3B	CTNNB1	CTNNB1
HSP90AB1	HSP90AB1	HSP90AB1	HSP90AB1	HSP90AB1
NEDD8	NEDD8	NEDD8	NEDD8	NEDD8
RPLP0	RPLP0	RPLP0	RPLP0	RPLP0
UBB	UBB	UBB	UBB	UBB
UBC	UBC	UBC	UBC	UBC

Supplemental Figure S1

Supplemental Figure S1

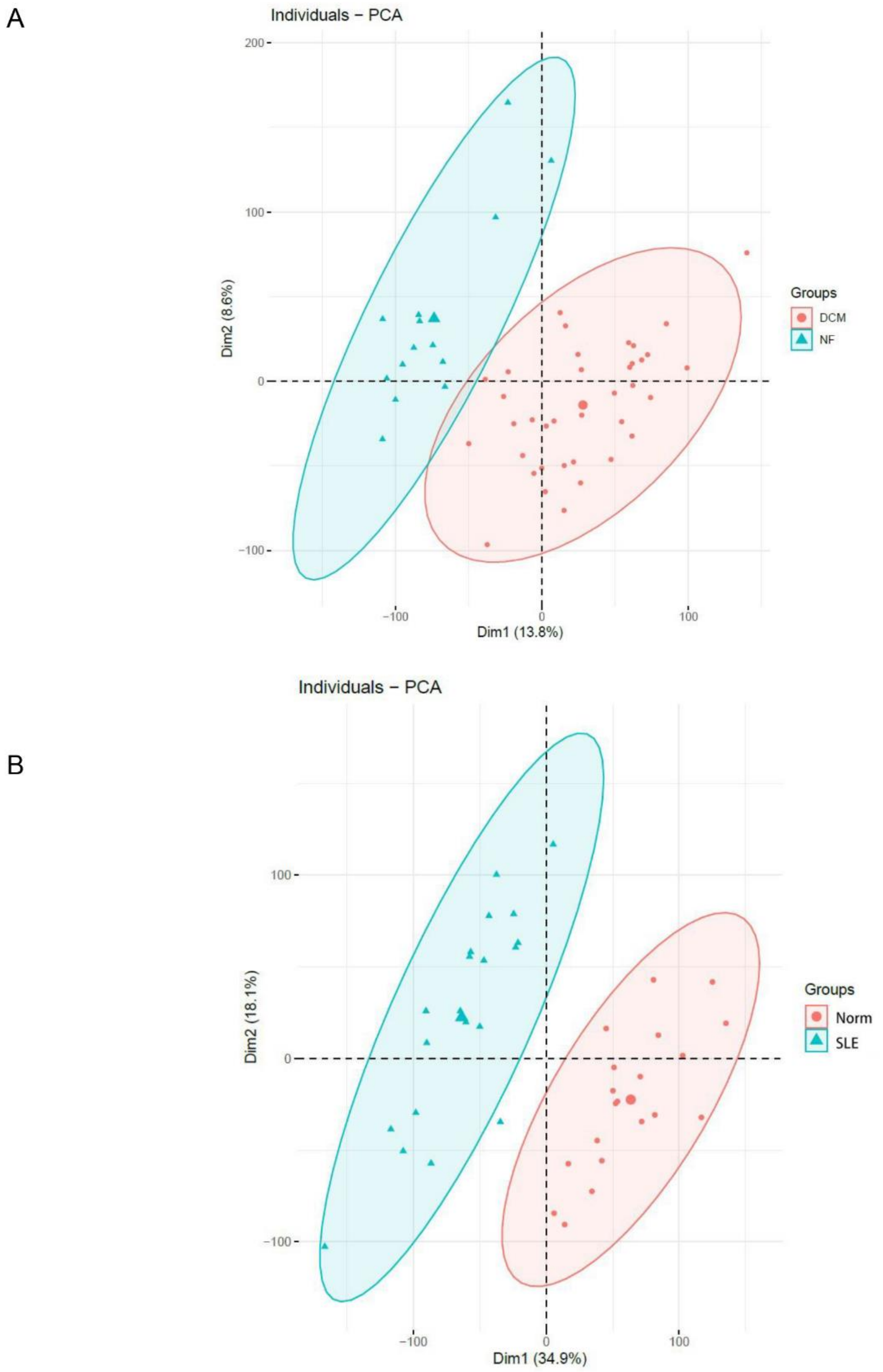

Supplemental Figure S2

Supplemental Figure S2

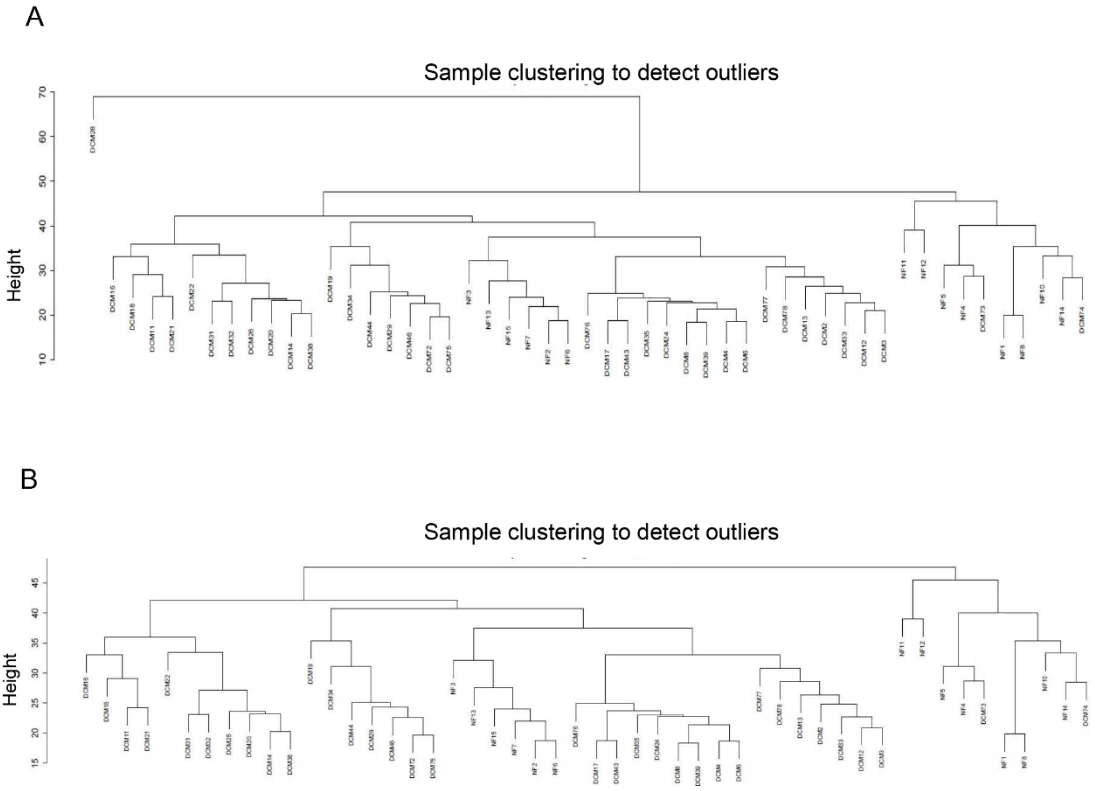

Figure legends

Supplemental Figure S1. The quality control of training datasets was performed through PCA clustering analysis. (**A**) PCA clustering analysis of HF dataset. (**B**) PCA clustering analysis of SLE dataset.

Supplemental Figure S2. Outlier clustering analysis on HF samples. (**A**) Cluster tree before removing the outliers. (**B**) Cluster tree after removing the outliers.
